# Dynamic TI for late gadolinium enhancement imaging in atrial fibrillation

**DOI:** 10.1186/1532-429X-14-S1-P264

**Published:** 2012-02-01

**Authors:** Jennifer Keegan, Peter D Gatehouse, Sonya V Babu-Naryanan, Rick Wage, David N Firmin

**Affiliations:** 1 Royal Brompton Hospital, London, UK

## Background

There has been considerable interest in high resolution 3D late gadolinium enhancement (LGE) imaging in the atrial fibrillation (AF) population, both pre and post RF ablation. Unlike conventional studies, these are performed with single R-wave gating to reduce the acquisition duration and the effects of gadolinium wash-out. For AF, the heart rate variability results in variable magnetization recovery between sequence repeats which, with a fixed inversion time (TI), causes ghosting and poor nulling of normal tissue. This is exacerbated by missed triggers and the number of poor quality studies is high. An adaptive inversion recovery preparation has previously been demonstrated in a 2D phantom acquisition [1]. The purpose of this study is to implement such a technique (dynamic_TI) for improved imaging in the AF population.

## Methods

An inversion-prepared segmented FLASH sequence was modified so that the inversion time was varied automatically from beat-to-beat based on the time since the last sequence repeat [2]. The gating delay was also modified for each cardiac cycle so that the timing of the acquisition window remained fixed. The sequence was demonstrated in a phantom mimicking a short axis slice of the myocardium and in patients with fast AF. Acquisitions were performed with single R-wave gating, both with and without the dynamic_TI algorithm. For phantom studies, both 2D and 3D data were acquired, while in vivo, time constraints only allowed the acquisition of 2D studies. The sequence repeat times were stored for subsequent simulation of the evolution of the longitudinal magnetisation through the acquisition.

## Results

**Figure 1:**
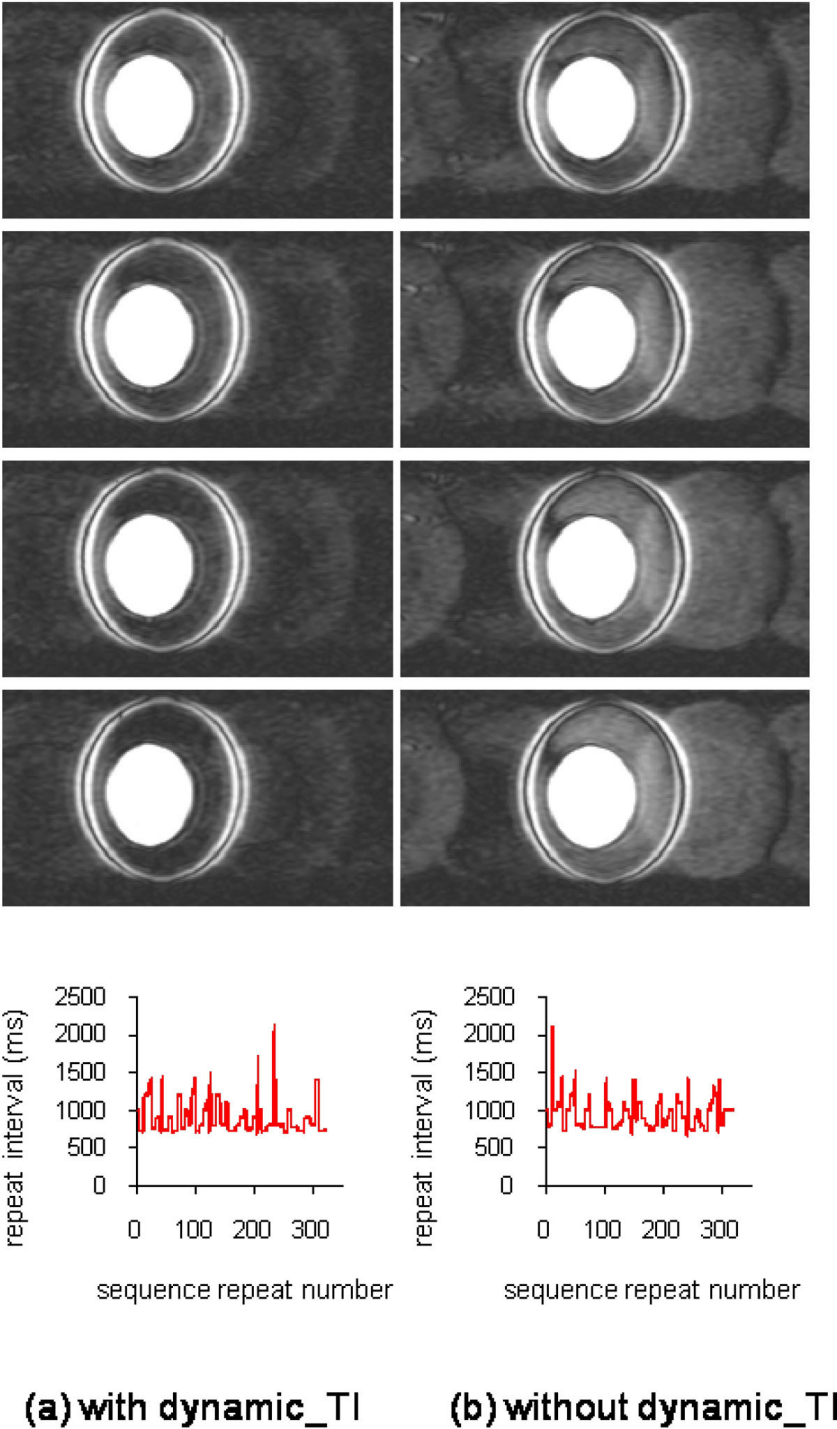
Four contiguous slices from 3D phantom acquisitions both with (left) and without (right) the dynamic_TI algorithm (brightness and contrast adjusted to show ghosting in both in-plane and through plane phase encoding directions). The sequence repeat time intervals throughout the acquisitions are shown below. The nulling is better with dynamic_TI and the images have much less ghosting.

**Figure 2:**
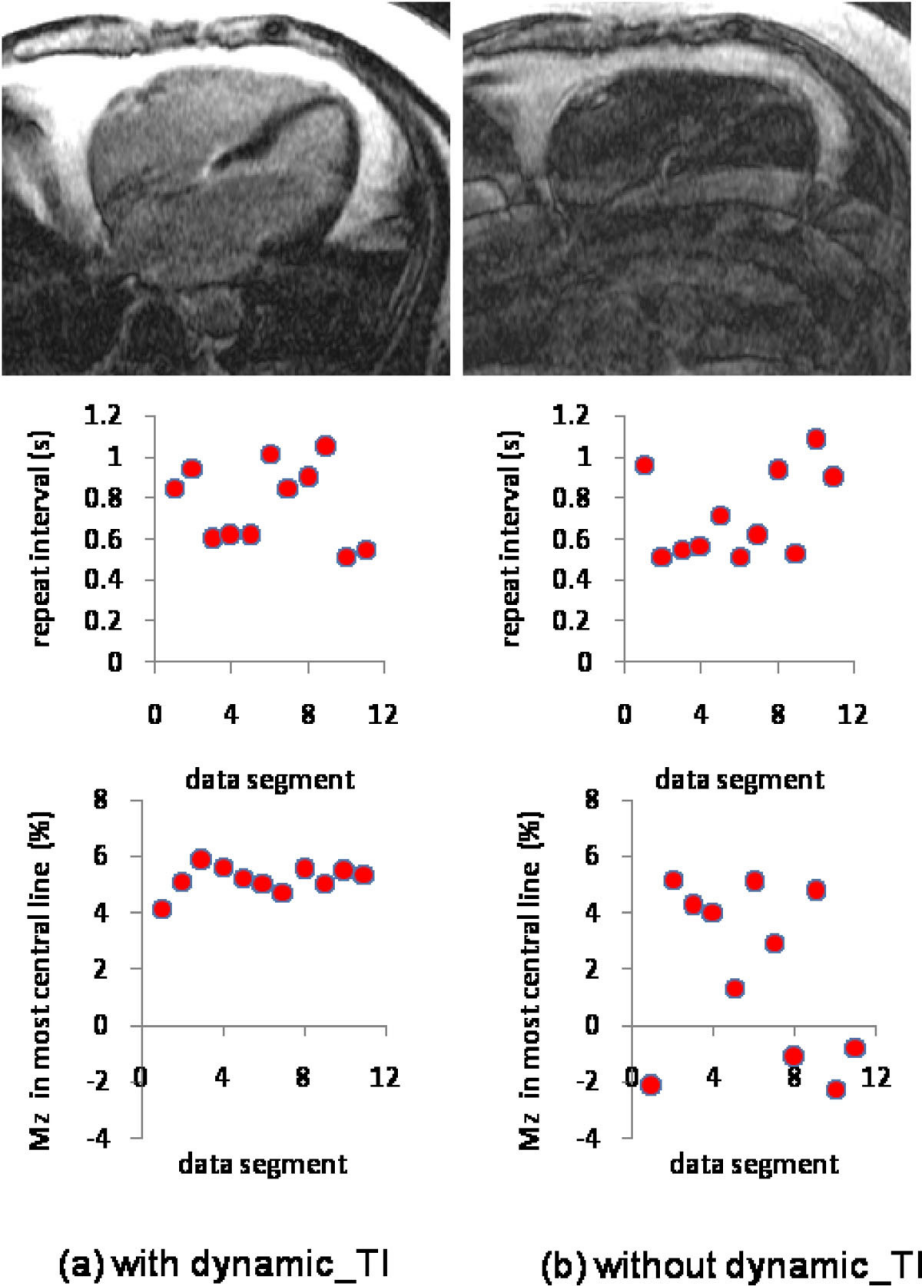
Transverse 2D LGE acquisitions in a patient with fast AF both with (left) and without (right) the dynamic_TI algorithm. The sequence repeat intervals throughout the acquisitions are also shown (middle), together with simulations of the longitudinal magnetisation (as a % of M0) for the central phase encode lines in each data segment. The average RR interval for this subject was ~500ms but missed triggers resulted in the average sequence repeat time interval being ~700ms in both cases. The dynamic_TI algorithm results in reduced longitudinal magnetisation variation throughout the acquisition and consequently, much better image quality.

Figure 1 shows a 3D phantom acquisition both with (left) and without (right) the dynamic_TI algorithm, together with plots of the sequence repeat time intervals throughout each 5minute acquisition. The variability in the repeat time intervals was similar for both acquisitions (912+/-226ms vs 959+/-204ms), with the dynamic_TI algorithm resulting in much less ghosting and better suppression. Figure 2 shows a 2D acquisition in a patient with fast AF which resulted in frequent missed triggers, together with the data segment repeat time intervals throughout each acquisition and the simulated longitudinal magnetisation for the most central phase encode lines in each segment. The dynamic_TI algorithm has resulted in minimal ghosting and good normal myocardial suppression despite highly variable sequence repeat time intervals. The simulations show that the dynamic_ TI algorithm has minimised longitudinal magnetisation variations throughout the acquisition. The TIs implemented in this acquisition ranged from 185 - 263ms.

## Conclusions

We have shown that dynamic adaptation of the inversion time for each cardiac cycle is feasible and results in less ghosting and improved nulling of normal myocardium in 2D acquisitions in the AF population. Application to 3D studies should result in improved acceptability rates.

## Funding

Wellcome Trust(grant reference: P32451) and NIHR Cardiovascular Biomedical Research Unit, Royal Brompton Hospital.
